# Prognostic value of poorly differentiated clusters in invasive breast cancer

**DOI:** 10.1186/1477-7819-12-310

**Published:** 2014-10-12

**Authors:** Ying Sun, Fenli Liang, Wei Cao, Kai Wang, Jianjun He, Hongyan Wang, Yili Wang

**Affiliations:** Center for Cancer Research, Department of Pathology, the First Affiliated Hospital of Xi’an Jiaotong University, 710061 Xi’an, China; Department of Pathology, School of Medicine, Xi’an Jiaotong University, Xi’an, China; Department of Pathology, Xi’an Medical College, Xi’an, China; Department of Oncology, Shaanxi Provincial People’s Hospital, Xi’an, China

**Keywords:** Breast cancer, Poorly differentiated cluster, Prognosis

## Abstract

**Background:**

Our study aimed to assess the prognostic value of poorly differentiated clusters (PDCs) in invasive breast cancer.

**Methods:**

A total of 146 cases of operable invasive ductal carcinoma that was not otherwise specified (IDC-NOS), from 2002 to 2009, were pathologically reviewed. Cancer clusters with five or more cancer cells and lacking gland-like structures were counted from a field containing maximum clusters in H & E slides under a × 20 objective lens (0.950 mm^2^ field of vision).

**Results:**

Tumors with <5, 5 to 9, and ≥10 clusters were graded as G1, G2, and G3, respectively (*n* =41, 60, and 45 tumors, respectively). An interobserver test showed good reproducibility, with a Cohen’s kappa coefficient of 0.739. The PDC grade was significantly associated with N stage (*P* <0.001), lymphovascular invasion (*P* =0.007), tumor budding grade (*P* <0.001), relapse rate (*P* <0.001), and death rate (*P* <0.001). Survival analyses revealed that the PDC grade was a significant prognostic factor for disease-free survival (hazard ratio 3.811; *P* <0.001) and overall survival (hazard ratio 3.730; *P* =0.001), independent of T stage, N stage, or tumor budding grade.

**Conclusions:**

The PDC grade is an independent prognostic factor of IDC-NOS. Considering the simplicity and availability of this method relative to conventional clinical pathology, PDCs may serve as a novel prognostic histological characteristic in IDC-NOS.

## Background

In general, tumor size, nuclear grade, mitotic activity, lymphatic and vascular invasion, and lymph node involvement are common clinical pathological features of breast cancer that can be detected by routine light microscopy. These parameters associated with the grading and staging of breast cancer are helpful in cancer diagnosis and prognostic assessment [1–3] However, determination of these factors cannot always accurately predict the biological characteristics of a tumor and the clinical outcome [4]. For example, a survival difference was observed clinically in patients with the same stage who had received similar clinical treatment [5], and more than 30% of patients with early-stage breast cancer have recurrent disease after effective therapy [6]. Variation in survival difference and clinical outcome partly suggests that standardized pathological factors do not perfectly reflect tumor aggressiveness and risk of recurrence and may even provide conflicting information about a patient’s prognosis [7]. The management of patients with breast cancer could benefit from the use of additional hallmarks as a supplement to conventional assessment.

Tumor budding is a pathological morphologic candidate index that has been applied to evaluate the prognosis of colorectal cancer [8, 9], breast cancer [10], and other cancers [11–13]. Tumor budding was defined as an isolated single cancer cell or a microscopic cluster with fewer than five cancer cells at the invasive frontal region of the tumor. High intensity tumor budding reflects malignant progression and is a promising prognostic factor for low survival rate. Tumor budding is considered to be related to the biological processes of cancer invasion and metastasis and was also postulated as the histological representation of epithelial mesenchymal transition [14]; it was recommended for inclusion in the reporting of colorectal cancer because of its significant prognostic value [15]. However, the use of tumor budding as a prognostic factor has limitations: budding can be observed only in the actively invasive frontal region; identifying tumor budding is difficult for single cancer cells or fairly small cell clusters in routine sections [16].

Poorly differentiated clusters (PDCs), a novel histopathologic indicator, provide additional tumor bioinformation in addition to tumor budding. These are cancer clusters composed of five or more cancer cells and lacking gland-like structures. The number of PDCs is highly relevant to survival and the incidence of nodal involvement in invasive colorectal cancer. A grading system based on PDCs successfully stratifies colorectal cancer cases by survival outcome and is believed to be useful in determining therapeutic strategies [17, 18]. Compared with tumor budding, counting larger clusters (≥5 cancer cells) in the whole tumor tissue stained with H & E is a sufficiently easy process [18]. So far, the description and prognostic value of the PDC grading of breast cancer has not been explored. Accordingly, we evaluated the prognostic value of PDC grading in 146 patients with invasive ductal carcinoma, that was not otherwise specified (IDC-NOS), which is the most common histological type of invasive breast cancer, and determined the relationship between PDC grading and other known prognostic parameters.

## Methods

### Case selection and clinicopathological review

Our study was approved by the Ethics and Research Committee of Shaanxi Provincial People’s Hospital. We retrieved patient information from the patient medical records room of Shaanxi Provincial People’s Hospital. We reviewed all hospital records, such as inpatient records, operative records, and outpatient clinic records. To avoid statistical deviation from different results for different tumor tissue types, we selected only IDC-NOS as the targeted object. Patients were excluded from the study if: (1) they presented with bone or distant spread at the time of primary cancer resections; (2) they received preoperative therapy; (3) H & E stained slides were unavailable for review. The survey included 146 female patients with IDC-NOS who underwent curative resections from January 2002 to December 2009. A total of 118 patients (80.8%) received systemic adjuvant therapies. Follow-up data were collected until death or August 2012, with a median follow-up period of 46 months (range, 4 to 112 months). A total of 44 (30.1%) cases showed relapse, and 31 patients (21.2%) died of tumor progression. Overall survival was defined as the interval between the date of operation and death from any cause. Disease-free survival was calculated from the date of the first surgery to the date of the local or systemic relapse or death resulting from any cause, whichever occurred first.

Clinicopathological data were obtained from recruiting records or by reviewing the archival H & E slides. The median age of the patients was 52 years (27 to 84). The histological grading was G1 in 7 cases (4.8%), G2 in 102 cases (69.9%), and G3 in 37 cases (25.3%), on the basis of the Bloom-Richardson system and the modification proposed by Elston and Ellis. According to the criteria of the TNM (tumor, node, metastases) system of the American Joint Committee on Cancer, the staging of tumor size was T1 in 24 cases (16.4%), T2 in 105 cases (71.9%), and T3 in 17 cases (11.6%). The presence of lymphovascular invasion within the primary tumor was identified in 35 cases (24.0%). The staging of node status was N0 in 70 cases (47.9%), N1 in 44 cases (30.1%) with one to three positive lymph node involvement and N2 or 3 in 32 cases (22.0%) with four or more positive. At the time of primary diagnosis, no patients presented with distant metastasis. The expression statuses of estrogen receptor, progesterone receptor, and HER-2, as determined by immunohistochemical analysis were collected by reviewing pathological reports. Cases were deemed positive for expression of estrogen receptor or progesterone receptor when at least 10% of the tumor cells had stained nuclei. Specimens with strong complete membranous staining in >30% of tumor cells were deemed positive for HER-2 overexpression. There were 57 cases (39.0%) with triple negative status.

### Definition and assessment of poorly differentiated clusters

Cancer clusters composed of five or more cancer cells and lacking gland-like structures were defined as PDCs. Using an Olympus microscope (BX-51), the entire tumor, including its advancing edge, was first scanned at a lower power magnification, to identify the five densest PDC areas. Subsequently, the clusters were counted under the microscopic field of a × 20 objective lens (field size 0.95 mm^2^), and the highest count of five areas per case was used as the number of PDCs. Tumors with fewer than five, five to nine, and ten or more clusters were graded as G1, G2, and G3, respectively [18]. Interobserver agreement was achieved for two independent observers (YS and KW). For both observers, this study was the first time they assessed PDCs. Discordances between the observers were resolved by a simultaneous review using a multihead microscope.

### Definition and assessment of tumor budding

Tumor budding was determined as a single cancer cell or as cancer clusters with fewer than five cancer cells at the invasive front. To determine the degree of tumor budding, like the counting method of PDCs, the clusters were counted under the × 20 objective lens in a field where budding was most intensively distributed. Tumors with fewer than five, five to nine, and at least ten budding foci were classified as G1, G2, and G3, respectively [18].

### Statistical analysis

Data were analyzed using the statistical package SPSS. Intra-observer variability was analyzed using Cohen’s kappa coefficient. Correlation between PDC grade and other clinicopathological variables were determined by the chi-square test or Fisher’s exact test. The Kaplan-Meier method and log-rank test were used in the analysis and comparison of survival curves. Univariate and multivariate survival analyses were carried out using Cox proportional hazards models. All analyses were two-sided, and a *P* value of less than 0.05 was considered statistically significant.

## Results

### Correlation between poorly differentiated clusters and other clinical pathological parameters

**Figure 1:**
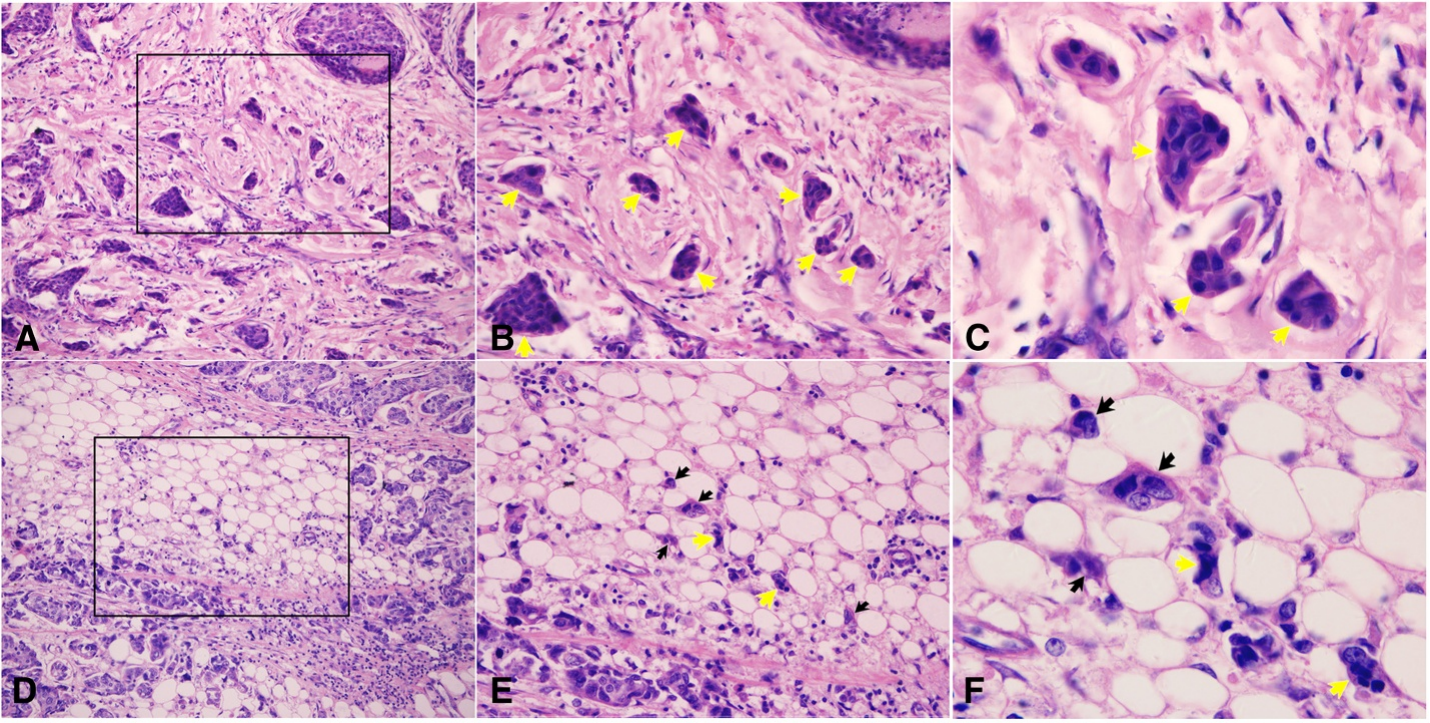
**Histologic findings of poorly differentiated cluster and tumor budding of IDC-NOS.** Cancer nests in the stroma, which do not show gland-like structures and are composed of up to five cancer cells, were defined as PDCs (indicated by yellow arrows. **(A)** 20×; **(B)** 40×; **(C)** 100×). Single cancer cells or clusters of fewer than five cancer cells were defined as tumor budding (indicated by black arrows. **(D)** 20×; **(E)** 40×; **(F)** 100×). Both PDC grade and tumor budding are determined from histological findings **(E,F)**.

Both PDC grade and tumor budding were histological findings in terms of loss of gland formation (Figure 1). Poorly differentiated clusters often appear within a tumor or at the advancing edge (Figure 1A,B,C). According to the number of PDCs, 41, 60, and 45 tumors were classified as G1, G2, and G3, respectively. The interobserver test showed good reproducibility, with a Cohen’s kappa coefficient of 0.739 (Table 1). The PDC grade was significantly associated with N stage (*P* <0.001), lymphovascular invasion (*P* =0.007), tumor budding grade (*P* <0.001), HER-2 overexpression (*P* =0.003), risk of relapse (*P* <0.001), and death (*P* <0.001) (Table 2). Other variables, such as age, histological grade, T stage, estrogen receptor or progesterone receptor expression, and triple negative status, were not significantly associated with the level of PDCs.

**Table 1:** **Results for the repeated observation of poorly differentiated clusters**

Second review	First review			
	G1	G2	G3	Total
G1	34	1	0	35
G2	8	48	4	60
G3	0	12	39	51
Total	42	61	43	146

Cohen’s kappa coefficient =0.739.

**Table 2:** **Correlation between poorly differentiated clusters and other clinicopathological characteristics**

Variable	Poorly differentiated clusters			***P***
	G1 (%)	G2 (%)	G3 (%)	
Age				
40 or younger	3 (7.3%)	11 (18.3%)	11 (24.4%)	0.103
>40	38 (92.7%)	49 (81.7%)	34 (75.6%)	
T stage				
T1	8 (19.5%)	10 (16.7%)	6 (13.3%)	0.779
T2	30 (73.2%)	43 (71.7%)	32 (71.1%)	
T3	3 (7.3%)	7 (11.6%)	7 (15.6%)	
N stage				
N0	30 (73.2%)	29 (78.3%)	11 (24.4%)	<0.001*
N1	8 (19.5%)	20 (33.3%)	16 (35.6%)	
N2 or 3	3 (7.3%)	11 (18.3%)	18 (40.0%)	
Grade				
G1	3 (7.3%)	4 (6.7%)	0 (0.0%)	0.134
G2	25 (61.0%)	42 (70.0%)	35 (77.8%)	
G3	13 (31.7%)	14 (23.3%)	10 (22.2%)	
Lymphovascular invasion				
Negative	36 (87.8%)	48 (80.0%)	27 (60.0%)	0.007*
Positive	5 (12.2%)	12 (20.0%)	18 (40.0%)	
Estrogen receptor/progesterone receptor expression				
Negative	20 (48.8%)	24 (40.0%)	19 (42.2%)	0.674
Positive	21 (51.2%)	36 (60.0%)	26 (57.8%)	
HER-2 overexpression				
Negative	38 (92.7%)	51 (85.0%)	29 (64.4%)	0.003*
Positive	3 (7.3%)	9 (15.0%)	16 (35.6%)	
Triple negative status				
No	23 (56.1%)	36 (60.0%)	30 (66.7%)	0.593
Yes	18 (43.9%)	24 (40.0%)	15 (33.3%)	
Tumor budding				
G1	27 (65.9%)	27 (45.0%)	9 (20.0%)	<0.001*
G2	11 (26.8%)	24 (40.0%)	20 (44.4%)	
G3	3 (7.3%)	9 (15.0%)	16 (35.6%)	
Relapse				
No	37 (90.2%)	49 (81.7%)	16 (35.6%)	<0.001*
Yes	4 (9.8%)	11 (18.3%)	29 (64.4%)	
Death				
No	38 (92.7%)	51 (85.0%)	25 (55.6%)	<0.001*
Yes	3 (7.3%)	9 (15.0%)	20 (44.4%)	
Total 146	41	60	45	

*P <0.05.

### Prognostic significance of poorly differentiated clusters

**Figure 2:**
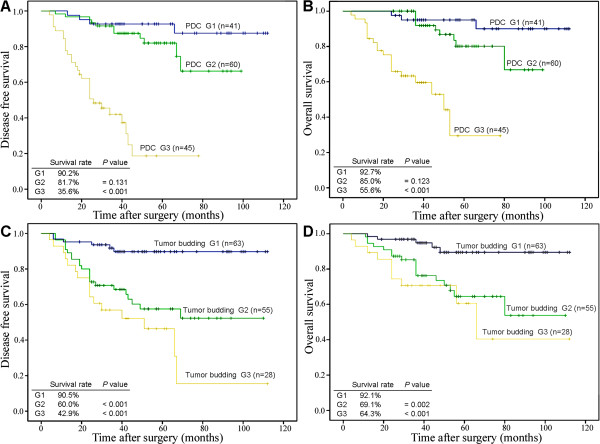
**Kaplan-Meier curves of disease-free survival and overall survival in IDC-NOS as a function of grade of poorly differentiated clusters and tumor budding.** Disease-free survival and overall survival differences were significant among the different PDC grade **(A,B)** and tumor budding **(C,D)** cohorts. (PDC, tumor budding: G3, G2 versus G1; log-rank test).

The Kaplan-Meier curves show that the disease-free survival rates were 90.2%, 81.7%, and 35.6% for PDC grades G1, G2, and G3, respectively. Similarly, the PDC grade correlated with the overall survival rate. The overall survival rates were 92.7%, 85.0%, and 55.6% for PDC grades G1, G2, and G3, respectively. Patients with PDC G3 had significantly worse disease-free survival and overall survival rates than those with G1 (*P* <0.001). The disease-free survival rate was 90.5% for G1 tumor budding, 60.0% for G2, and 42.9% for G3, whereas the overall survival rate was 92.1%, 69.1%, and 64.3% for G1, G2, and G3 tumor budding, respectively. Compared with patients with G1, patients with G2 and G3 tumor budding had significantly worse disease-free survival and overall survival rates (*P* <0.05) (Figure 2).

Univariate survival analysis revealed that PDC grade, age, T stage, N stage, lymphovascular invasion, HER-2 overexpression, and tumor budding grade were significantly associated with disease-free survival and overall survival (Tables 3 and 4). In multivariate Cox regression analysis, PDC grade (hazard ratio 3.811, *P* <0.001; hazard ratio 3.730, *P* =0.001), T stage (hazard ratio 3.135, *P* <0.001; hazard ratio 4.064, *P* =0.001), and N stage (hazard ratio 2.922, *P* =0.012; hazard ratio 3.482, *P* =0.023) were identified as the independent prognostic factor for disease-free survival and overall survival, respectively (Tables 3 and 4). Tumor budding grade (hazard ratio 1.808, *P* =0.009) was an independent prognostic factor only for disease-free survival (Table 3).

**Table 3:** **Disease-free survival analysis by the Cox proportional hazards regression model in 146 cases of IDC-NOS**

Variable	Univariate			Multivariate		
	Hazard ratio	95% confidence interval	***P***	Hazard ratio	95% confidence interval	***P***
Age 40 or younger/>40	0.501	0.257 to 0.975	0.042*	0.826	0.414 to 1.648	0.587
T stage: T1/T2/T3	2.554	1.451 to 4.497	0.001*	3.135	1.672 to 5.879	<0.001*
N stage: N0/N1/N2 or 3	2.795	1.921 to 4.064	<0.001*	2.922	1.266 to 6.744	0.012*
Grade: G1/G2/G3	1.455	0.829 to 2.554	0.191			Not assessed
Lymphovascular invasion: negative/positive	3.809	2.095 to 6.925	<0.001*	0.593	0.183 to 2.155	0.427
Estrogen receptor or progesterone receptor: negative/positive	1.009	0.555 to 1.834	0.978			Not assessed
HER-2: negative/positive	2.064	1.031 to 4.133	0.041	0.820	0.383 to 1.758	0.610
Triple negative status: no/yes	1.365	0.731 to 2.551	0.329			Not assessed
Poorly differentiated cluster grade: G1/ G2/ G3	5.086	2.998 to 8.628	<0.001*	3.811	1.992 to 7.289	<0.001*
Tumor budding: G1/G2/G3	2.554	1.721 to 3.792	<0.001*	1.808	1.159 to 2.820	0.009*

*P <0.05.

**Table 4:** **Overall survival analysis by the Cox proportional hazards regression model in 146 cases of IDC-NOS**

Variable	Univariate			Multivariate		
	Hazard ratio	95% confidence interval	***P***	Hazard ratio	95% confidence interval	***P***
Age 40 or younger/>40	0.410	0.193 to 0.869	0.020*	0.622	0.279 to 1.388	0.246
T stage: T1/T2/T3	2.956	1.513 to 5.775	0.002*	4.064	1.839 to 8.982	0.001*
N stage: N0/N1/N2 or 3	2.890	1.848 to 4.519	<0.001*	3.482	1.185 to 10.234	0.023*
Lymphovascular invasion: negative/positive	3.841	1.909 to 7.729	<0.001*	0.516	0.106 to 2.507	0.412
Grade: G1/G2/G3	1.698	0.880 to 3.276	0.114			Not assessed
Estrogen receptor or progesterone receptor: negative/positive	0.922	0.460 to 1.849	0.819			Not assessed
HER-2: negative/positive	2.577	1.172 to 5.668	0.019*	0.874	0.357 to 2.136	0.767
Triple negative status: no/yes	1.393	0.670 to 2.895	0.375			Not assessed
Poorly differentiate cluster grade: G1/G2/G3	4.987	2.681 to 9.276	<0.001*	3.730	1.718 to 8.101	0.001*
Tumor budding: G1/G2/G3	2.285	1.442 to 3.623	<0.001*	1.517	0.597 to 2.564	0.120

*P <0.05.

## Discussion

Pathologists are expected to provide complete information on histological features, such as tumor description, orientation, and analysis of surgical margins [19]. In clinical practice, Elston’s modified Bloom and Richardson method is a widely accepted tumor histological grading system with good prognostic correlation. Histological grade forms part of the multifactorial Nottingham prognostic index, together with tumor size and lymph node stage. The Nottingham prognostic index is used to deliver appropriate therapy to individual patients [20, 21]. However, in practice, the grading system is not always optimal, for two reasons. Disparity and diagnostic variability between observers exists in routine pathological observation and diagnosis based on subjective opinion [19, 22]. Objectively, breast cancer is characterized by generous morphologic heterogeneity [23]. There is considerable variability in microscopic examination of histological growth pattern and cellular differentiation. Therefore, identification of less common histological patterns can provide clinically useful data [24]. For instance, budded tumor cells at the margin were regarded as a poorly differentiated component, which could illustrate aggressive cancer behavior and adverse prognosis [25, 26]. Grading and typing in breast cancer constitutes the major content of a pathological report. However, the minor but key poorly differentiated elements may determine clinical outcome [10]. As a necessary complement, novel morphologic parameters that can be determined with simplicity, objectivity, and reproducibility are required to display tumor biological features. To date, tumor budding, as a candidate index of tumor cell invasive potential, denotes the tumor feature in the marginal area instead of the entire tissue. Cytokeratin immunohistochemical staining is required to diagnose tumor budding accurately because identifying single cancer cells and fairly small cell clusters in routine sections is difficult. However, the required staining is considered a minor inconvenience [16]. The introduction of a new histopathological parameter, that is, PDCs, in 2008 (tumor cells ≥5) might compensate for these disadvantages [17]. In 2012, Ueno *et al.* also confirmed that PDCs, which are representative of potential tumor biological aggressiveness, can be used as robust prognostic markers [18]. The PDC-based grading system is expected to be less subjective and more informative for prognostic prediction, compared with conventional tumor grading systems [27], TNM staging in colorectal cancer [18, 28]. Identification and counting of PDCs in routine pathologic diagnosis is more simple and accurate than identifying tumor budding via immunohistochemical techniques [18].

In this study, we investigated the prognostic value of PDCs in IDC-NOS. The three main findings of this study are as follows. First, PDCs were confirmed as a significant prognostic factor independent of classical or recent pathological morphologic variables, such as tumor size, node status, and tumor budding. Second, a high PDC grade, representing high invasive potential, was associated with lymph node involvement, lymphovascular invasion, high tumor budding grade, and poor clinical outcome. Third, a good interobserver agreement was found in counting PDCs.

As poorly differentiated components, both PDCs and tumor budding are the result of histological findings in terms of loss of gland formation. Poorly differentiated clusters often appear within a tumor and at the advancing edge, whereas tumor budding is observed in the actively invasive frontal region [18]. In this study, a higher PDC grade (G2, G3) was significantly associated with tumor aggressive and invasive indexes, such as N stage, lymphovascular invasion, and higher tumor budding grade. A feasible explanation is that PDCs, like tumor budding, present the epithelial mesenchymal transition of neoplastic cells with acquired properties of tumor stem cells [26, 28–30]. The PDC grade is correlated with the prognosis of IDC-NOS patients, since a higher PDC grade (G2, G3) correlated with a high relapse and death rate, and PDC G3 exhibited shorter disease-free survival and overall survival times. A high PDC grade is a valuable morphologic parameter, indicating tumor invasive behavior and poor prognosis.

In our results, the PDC grade was not associated with estrogen receptor or progesterone receptor expression or triple negative status, but was associated with HER-2 overexpression. However, the mechanism underlying PDC grade and HER-2 overexpression is unclear. Considering that fewer than 30 patients with HER-2 overexpression were studied and different results exist between univariate and multivariate survival analyses on disease-free survival and overall survival, we presumed that an accidental error had occurred in the study. A large-scale sample is required to confirm the correlation between PDC grade and HER-2 overexpression.

In this study, PDCs and tumor budding were counted simultaneously, and their prognostic value was assessed; it was found that they shared common stratified standards, according to the findings of Ueno *et al.* [18]. Ueno *et al*. had confirmed that PDC grading is more powerful in assessing prognostic outcome than tumor budding in colorectal cancer [17, 18]. In this study, a similar result was found; that PDCs are an independent prognostic factor for both relapse and death of IDC-NOS patients, while tumor budding is an independent prognostic factor for relapse but not death. Indeed, multivariate Cox regression analysis of tumor budding in this study slightly disagreed with that of a previous study [10]. We believe that the difference between our results and those of the previous report was caused by a disparity in cut-off value: the cut-off value was five and ten, respectively, at three grades, instead of seven at two grades, as with the study of Liang *et al.* [10]. The change of the stratified standard led to a minimal deviation in the result. Nevertheless, Kaplan-Meier curves revealed that patients with G2 or G3 tumor budding had significantly worse overall survival compared with those with G1. It still strongly indicated high-level tumor budding is highly correlated with the poor prognosis in IDC-NOS.

More importantly, a Cohen’s kappa coefficient of 0.739 indicated a good agreement of PDC grade between two pathologists. The interobserver test in this study, as found in previous studies on colorectal cancer, demonstrates that PDC grade is a reliable and reproducible method with more objectivity for patients with IDC-NOS. With regard to the simplicity of PDC assessment based only on routine H & E section observation and PDC counting within the whole tumor tissue, PDCs can provide more morphological and prognostic information than conventional histology grade and tumor budding, with no need for additional techniques or costs.

The findings in this study disclosed the clinical significance of PDCs in IDC-NOS. A high PDC grading reflected aggressive behavior and adverse prognosis of the tumor. This new histological parameter could be used to complement traditional histopathological prognostic factors in breast carcinoma. However, the PDC molecular features remain unclear. Further exploration is necessary to elucidate the biological significance of PDCs.

## Conclusions

Our results confirmed that PDCs are a reproducible, significant, and independent prognostic factor in IDC-NOS. The fact that a simple cell-based parameter using conventional microscopy can possess such a high predictive power is remarkable. Poorly differentiated clusters can be viewed as morphologic candidate indexes in breast cancer. This study is only a preliminary investigation. Further work, such as validating prognostic value in larger samples, elucidating the underlying mechanism of PDCs, and testing PDCs in clinical practice, is warranted.

## Abbreviations

H & E: hematoxylin and eosin
IDC-NOS: invasive ductal carcinoma, not otherwise specified
PDC: poorly differentiated cluster
TNM: ‘tumor, node, metastases’.
